# Diversity and Comparison of Intestinal *Desulfovibrio* in Patients with Liver Cirrhosis and Healthy People

**DOI:** 10.3390/microorganisms11020276

**Published:** 2023-01-20

**Authors:** Gexue Lu, Yu Zhang, Yilin Ren, Jin-Song Shi, Zheng-Hong Xu, Yan Geng

**Affiliations:** 1 School of Life Sciences and Health Engineering, Jiangnan University, No. 1800 Lihu Avenue, Wuxi 214122, China; 2 Department of Gastroenterology, Affiliated Hospital of Jiangnan University, Wuxi 214122, China; 3 National Engineering Laboratory for Cereal Fermentation Technology, Jiangnan University, Wuxi 214122, China; 4 Jiangsu Engineering Research Center for Bioactive Products Processing Technology, Jiangnan University, Wuxi 214122, China; 5 The Key Laboratory of Industrial Biotechnology, Ministry of Education, School of Biotechnology, Jiangnan University, Wuxi 214122, China

**Keywords:** *Desulfovibrio*, gut microbiota, chronic liver disease, hydrogen sulfide, antibiotics, Sulfate-reducing bacteria

## Abstract

*Desulfovibrio* belongs to Sulfate-reducing bacteria (SRB), which are widely present in anaerobic environments, including the human gut. *Desulfovibrio* has been associated with many human diseases, including chronic liver disease. However, the characteristics and difference of *Desulfovibrio* from fecal samples of healthy volunteers (HV) and patients with liver cirrhosis (LC) have not been fully elucidated. Here, we isolated *Desulfovibrio* from the feces of 6 HV and 9 LC, and 88 *Desulfovibrio* strains were obtained. In the feces of HV, 55% of isolated strains were *D. desulfuricans*, followed by *D. intestinalis* (15%), D. *simplex* (11%), *D. piger* (9%), *D. legallii* (4%), *Cupidesulfovibrio oxamicus* (4%) and *D. fairfieldensis* (2%). However, only *D. desulfuricans* (60%) and *C. oxamicus* (40%) were isolated from fecal samples of patients with LC. Our results suggest that there was a significant difference in the desulfurization ability and the H_2_S production ability of different *Desulfovibrio*. *Desulfovibrio*. Furthermore, we found that *Desulfovibrio* isolated from the patients with LC generally had a higher hydrogen sulfide production capacity, gastrointestinal tolerance, and levels of antibiotic resistance than the same species isolated from HV. Our findings suggested that *Desulfovibrio* may be associated with the occurrence and development of liver cirrhosis.

## 1. Introduction

The term gut microbes refers mainly to a large number of microbiota living in the host’s intestinal tract. With the development of modern medicine, human gut bacteria have been regarded as a “silent organ”, which has been found to take an important role in maintaining normal physiological, biochemical, and immunomodulatory functions of the human body [1,2,3]. Accumulating evidence demonstrates that inflammatory bowel disease (IBD), non-alcoholic fatty liver disease (NAFLD), atherosclerosis, and other diseases have a symbiotic relationship with the imbalance of gut bacteria [4].

*Desulfovibrio* genus is a type of Sulfate-reducing bacteria (SRB) that widely exists in anaerobic environments, including soil, water, and the intestinal tract [5]. It plays a critical role in the global carbon and sulfur cycle. With the rapid development of modern bioinformatics technology and the in-depth study of intestinal microorganisms, the relationship between SRB and human diseases has been gradually understood and revealed. *Desulfovibrio* is the dominant SRB in healthy human gut microbiota, accounting for about 93% [6,7].

As the dominant population of intestinal SRB in humans, *Desulfovibrio* will affect the physiological metabolism of the liver due to the gut–liver axis [8]. Some studies have shown that *Desulfovibrio* increases significantly in liver diseases such as NAFLD and liver fibrosis [9,10]. The NAFLD activity score has been positively correlated with the relative abundance of *Desulfovibrio* [11]. An increased abundance of *Desulfovibrio* was found in feces of obese children with NAFLD compared to those without NAFLD, and intragastric gavage with *D. piger* increased intestinal permeability and aggravated liver steatosis and fibrosis in obese mice [12]. On the contrary, *D. vulgaris* was enriched in *Astragalus* polysaccharide-treated, high-fat-diet-fed mice, which could produce acetic acid and showed an anti-NAFLD effect [13].

In addition to the direct influence of *Desulfovibrio*, its metabolites are also the key factors leading to the emergence and development of intestinal diseases, prompting the body to release inflammatory factors, such as Interleukin-6 (IL-6), Interleukin-8 (IL-8), etc., thus affecting the health of the body [14]. Hydrogen sulfide (H_2_S) is one of its main metabolites, and it can inhibit butyrate oxidation and has cytotoxicity at a high concentration [15]. The effects of H_2_S on the liver are complex and varied. Exogenous H_2_S can promote the proliferation of hepatic stellate cells and the expression of hepatic fibrosis markers [16]. However, NaHS (a donor of H_2_S) supplementation was found to ameliorate liver fibrosis in carbon tetrachloride-induced rodents models [17,18].

Previous studies have shown that *Desulfovibrio* may play a role in the occurrence and development of liver disease, but there were differences at the species level. We speculate that the diversity and difference of *Desulfovibrio* isolated from the human gut may affect their physiological functions. In this study, we isolated and purified the different *Desulfovibrio* from the gut of healthy volunteers (HV) and patients with liver cirrhosis (LC). A series of comparative studies, including desulfurization capability, H_2_S production capacity, intestinal colonization capability, and antibiotic sensitivity, were carried out. The results can provide some reference basis for further exploring the relationship and mechanism between *Desulfovibrio* and liver cirrhosis.

## 2. Materials and Methods

### 2.1. Sample Collection

Fecal samples were collected from six healthy volunteers and nine patients with liver cirrhosis. The patients had cirrhosis induced by factors other than alcohol. Six healthy volunteers met the following criteria: none of them took antibiotics within two months before sampling, and none of them had a history of gastrointestinal and liver disease. Nine patients met the following criteria: none of them took antibiotics within two months before sampling. The feces samples were collected and stored at 4 °C and transferred to the laboratory within 2 h. All operating procedures were approved by the Medical Ethics Committee of Wuxi Second People’s Hospital (Approval number: No. 20170608). All subjects gave their informed consent for inclusion before they participated in the study.

### 2.2. Culture Maintenance

*Desulfovibrio desulfuricans* (ATCC 29577) was obtained from the American Type Culture Collection. For all experiments and culture maintenance, we used the EDGM medium [19]. One liter of EDGM medium (pH 7.8) contains 0.5 g KH_2_PO_4_, 1.0 g Na_2_SO_4_, 1.0 g NH_4_Cl, 0.05 g CaCl_2_, 2.0 g MgCl_2_·6H_2_O, 5.0 g CH_3_COONa, 1.0 g yeast extract, 1.0 g sodium pyrosulfite, 1.0 g FeSO_4_·7H_2_O, 0.1 g vitamin C, 0.1 g sodium thioglycolate, 5 mg vancomycin, 10 mg colistin sulphate, and 1 mg resazurin. Solid medium was supplemented with an additional 15.0 g agar.

All cultures were grown at 37 °C under anaerobic conditions (10% Hydrogen, 10% Carbon dioxide, and 80% Nitrogen). The tubes with strain were brim-filled with medium and closed to provide anaerobic conditions. All strains were stored in EDGM media with 30% glycerol at −80 °C.

### 2.3. Enrichment and Biochemical Characteristics of the Strain

An amount of 1 g of each fecal sample was blended with PBS and vortexed thoroughly. The mixture was centrifuged to separate the bacteria from organic particles. A 10% inoculation amount was inoculated in 30 mL EDGM medium, then the anaerobic culture was carried out at 37 °C for four days and then transferred three times [20]. Black precipitation (FeS) appeared in the culture system, which was accompanied by the smell of rotten eggs (H_2_S), and H_2_S could be detected at the mouth of the culture bottle with lead acetate test paper, which indicated that *Desulfovibrio* was enriched successfully. Enrichment of *Desulfovibrio* was serially diluted 10-fold, and 200 μL of the dilutions (10^−4^, 10^−5^, and 10^−6^) were plated onto EDGM agar. All cultures were grown at 37 °C for 5~7 d under anaerobic conditions (10% Hydrogen, 10% Carbon dioxide, and 80% Nitrogen). The round colonies with a black surface were selected, and all colonies were picked and purified by restreaking onto EDGM agar at least thrice until single. 

The purified Isolates were observed by optical microscopy to examine morphology and Gram-negative status. Additionally, catalase activity, Voges Proskauer test (V-P test), and Methyl Red test (M.R. test) were carried out as described [19,21]. The potential *Desulfovibrio* isolates that tested Gram-negative, catalase-positive, V-P test-negative, and M.R. test-negative were preserved in EDGM media with 30% glycerol at −80 °C.

### 2.4. Isolate Identification and 16S rRNA Phylogenetic Trees

Total DNA was extracted using a Rapid Bacterial Genomic DNA Isolation Kit (Generay Biotech, China), according to the manufacturer’s protocol. The 16S rRNA gene fragments were PCR amplified using the barcoded adaptor-containing forward 27F (5′-AGA GTT TGA TCC TGG CTC AG-3′) and reverse 1492R (5′-GGT TAC CTT GTT ACG ACT T-3′) primers. The products were sequenced using Sanger sequencing by 3730xl DNA Analyzer at Tianlin Biotechnology, Wuxi. Then, the sequence was blasted against NCBI Ref Seq for taxonomic identification, and isolates were identified with the highest bit score [22]. To generate phylogenetic trees, 16S rRNA gene sequences were aligned using ClustalW. BIONJ was used to generate the starting tree, and the nearest neighbor interchange (NNI) was used for tree improvement. The ClustalW alignment and MEGA analysis were performed without specifying any taxonomy or an outgroup, and subsequently, the resulting dendrogram was rooted on *Desulfovibrio* and *Cupidesulfovibrio* type strains and isolates for presentation using Ggtree. These results were calculated using bootstrap resampling with 5000 replications. The sequences were deposited in the NCBI under the accession number OQ154877~OQ154964.

### 2.5. Desulphurization Performance Evaluation

The evaluation was mainly carried out from two aspects: the pH of the culture system and the change of the clearance rate of SO_4_^2−^ in the culture system. All strains were cultured at 37 °C for four days under anaerobic conditions. The pH and the clearance rate of SO_4_^2−^ in the culture system were measured at day 0 and day 4, as described previously [23]. The clearance rate was measured by gravimetry. An amount of 10 mL culture solution of bacteria which was isolated from fecal samples was filtered by 0.45 μm filter. Then, 50 μL drop of (0.1%) methyl red indicator was added, and 0.2 mol/L hydrochloric acid solution or ammonia was used to adjust the solution to orange-yellow. After adding 0.2 mL concentrated hydrochloric acid, the solution was heated and boiled for 5 min. About 1 mL 100 mg/L BaCl_2_ hot solution was slowly added until precipitation no longer occurred. The precipitation was aged at 50~60 °C for six hours. The next steps were washing and precipitation in hot water, drying, cooling, and weighing.

### 2.6. Effect of Fe^2+^ on D. desulfuricans ATCC 29577

Using the Fe^2+^ concentration in the EDGM medium as the reference concentration (1.8 mM), a series of FeS suspensions was converted into ratio diluents (0.078125, 0.15625, 0.3125, 0.625, 1.25, 2.5, 5, 10 mM). The OD_600_ of them was detected, the relationship curve was drawn, and linear analysis was performed.

EDGM medium containing different Fe^2+^ concentrations of 0, 0.45, 0.9, 1.8, 3.6, 7.2, and 14.4 mM were prepared. *D. desulfuricans* ATCC 29577 was inoculated and cultured at 37 °C for four days under anaerobic conditions. Then, the pH and the clearance rate of SO_4_^2−^ in the culture system were measured.

### 2.7. Hydrogen Sulfide Metabolism

The H_2_S in the culture solution of bacteria isolated from fecal samples was detected by the H_2_S detection kit (Nanjing Jiancheng Biological Engineering Institute). The endogenous H_2_S release in living cells was measured by the colorimetric assay as previously described [24]. The 5% polyvinylpyrrolidone (PVP) solution and perfluorosulfonic acid resin (Nafion) solution were evenly mixed according to the volume ratio at 9:1, and the AgNO_3_ solution of 0.1 M was added to prepare the PVP-Nafion-AgNO_3_ mixture, which was placed away from light at room temperature for 24 h, waiting for the bubble to dissipate completely. In total, 15 μL PVP-Nafion-AgNO_3_ mixed droplets on the inside of the 96-well plate and dried at room temperature for at least 1 h until the film was formed. In total, 200 μL of Sodium sulfide solutions (12.5, 25, 50, 125, 150 μM) was used to draw the standard curve. *D. desulfuricans* ATCC 29577 and *Desulfovibrio* isolated were inoculated into a 96-well plate for 24 h. The OD_310_ was detected, and the yield of H_2_S was calculated by standard curve.

### 2.8. Gastrointestinal Tolerance

*D. desulfuricans* ATCC 29577 and the isolated strains were inoculated into the gastric fluid (SGF) or simulated intestinal fluid (SIF) [25], and then the anaerobic culture was carried out at 37 °C. The OD_600_ was detected at 0, 0.5, 1.0, 1.5, 2.0, 2.5, and 3.0 h. The survival condition of the strain in SGF and SIF was observed to determine their tolerance to the gastrointestinal environment.

### 2.9. Antibiotic Resistance

These assays, which were based on a previously published method [26], were performed. The initial OD_600_ values of *D. desulfuricans* ATCC 29577 and isolated strains were adjusted to about 0.4, and 10% of the inoculum was inoculated into the fresh medium containing different antibiotics. The following antibiotics were tested: Neomycin sulphate (40 μg/mL), Polymyxin B sulphate (25 μg/mL), Nisin (25 μg/mL), Ampicillin (25 μg/mL), Metronidazole (20 μg/mL), Kanamycin sulphate (20 μg/mL). The evaluation criteria of resistance were referred to as EUCAST (European Committee on Antimicrobial Susceptibility Testing). *D. desulfuricans* ATCC 29577 was included as a control. The culture medium without antibiotics was considered the blank group, and the medium without inoculation as the blank control group was cultured statically under anaerobic conditions at 37 °C. The growth curve was constructed according to the OD_600_ value measured at 0, 1, 2, 4, 8, 12, 16, 24, 30, 36, 48, 60, 72, 84, 96, 108, 120, 132, 144 h. Additionally, different concentrations of antibiotics (0.000, 0.125, 0.250, 0.500, 0.750, 1.000, 1.250, 2.500, 5.000, 7.500, 10.00, 12.50, 15.00, 20.00, 25.00, 30.00, 35.00, 40.00, 45.00, 50.00 μg/mL) were used to detect the minimum inhibitory concentration (MIC). OD_600_ value was measured at 0, 12, 24, 48, and 72 h.

### 2.10. Statistical Analysis

Statistical analyses were carried out with SPSS for Windows V22.0 (SPSS Inc., Chicago, IL, USA). One-way ANOVA followed by Dunnett’s multiple comparisons test was used for multiple comparisons (GraphPad Prism, San Diego, CA, USA), and differences between two groups were tested by independent samples *t*-tests. Data are expressed as the mean ± standard error. Significance was set at *p* < 0.05.

## 3. Results

### 3.1. Identification and Phylogeny of Desulfovibrio Isolates

**Figure 1:**
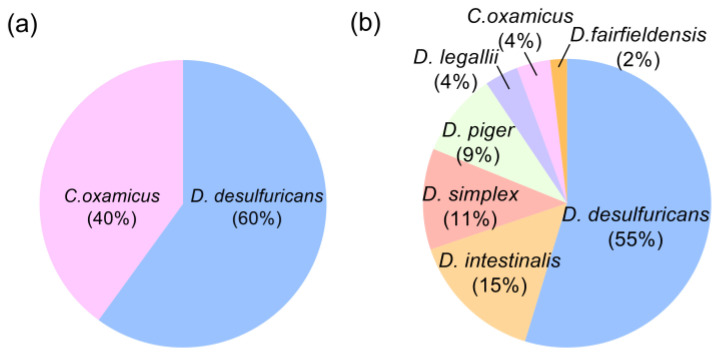
Percentage of different *Desulfovibrio* strains from gut of (**a**) patients with liver cirrhosis (Total = 35) and (**b**) healthy volunteers (Total = 53).

To characterize the diversity within the *Desulfovibrio* genus, we collected stool samples from 15 human donors, including 6 healthy volunteers (HV) and 9 patients with liver cirrhosis (LC) (Table S1). A total of 255 possible *Desulfovibrio* strains were obtained from the 12 stool samples, including 164 strains from healthy volunteers and 92 strains from patients with liver cirrhosis. These strains were all Gram-negative, V-P test-negative, M.R. test-negative, and catalase-positive. Based on 16S rRNA gene sequence analysis, we identified 53 strains from HV and 35 strains from LC belonging to *Desulfovibrio* (Table S2). Strains of LC were *Desulfovibrio desulfuricans* (*D. desulfuricans*) (60%) and *Cupidesulfovibrio oxamicus* (*C. oxamicus, Desulfovibrio oxamicus*) (40%) (Figure 1a). *C. oxamicus* was a member of *Desulfovibrio* before a recent study [27], and there were few studies on the properties of this species in the human gut. Our results showed that the physiological characteristics of *C. oxamicus* were similar to those of *Desulfovibrio*, so we compared *C. oxamicus* in this study. The majority of HV sources were *D. desulfuricans*, which accounted for 55% of the total isolated strains, followed by *Desulfovibrio intestinalis* (*D. intestinalis*) (15%), *Desulfovibrio simplex* (*D. simplex*) (11%), *Desulfovibrio piger* (*D. piger*) (9%), *Desulfovibrio legallii* (*D. legallii*) (4%), *C. oxamicus* (4%), and *Desulfovibrio fairfieldensis* (*D. fairfieldensis*) (2%) (Figure 1b). For the follow-up experiment, we named the strains isolated from HV as *D. desulfuricans* JN-1, -2, -6, -8, -10, -11, -13, -15, -16, -22, -25, -27, -28, -29, -30, -31, -33, -34, -35, -38, -39, -41, -42, -43, -46, -48, -51, -52, and -53; *D. intestinalis* JN-3, -4, -7, -9, -23, -37, -49, and -50; *D. simplex* JN-17, -19, -24, -26, -36, -47; *D. piger* JN-12, -18, -20, -21, -44; *D. legallii* JN-32, -40; *C. oxamicus* JN-5, -14; and *D. fairfieldensis* JN-45. The isolations from LC were named *D. desulfuricans* JN-B1~B20, and *C. oxamicus* JN-B21~B35.

**Figure 2:**
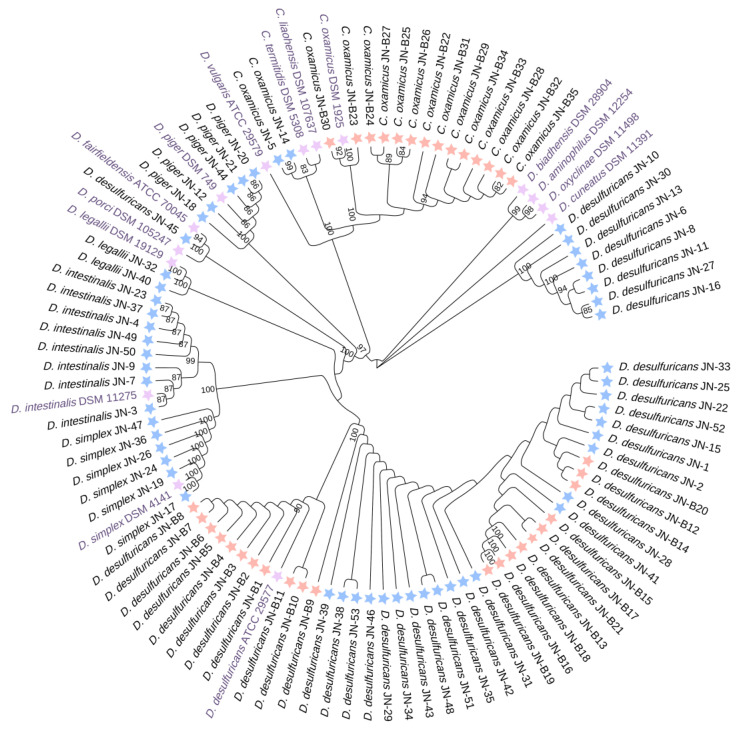
Phylogenetic analysis dendrogram of relationship sequences of 16S rRNA gene of *Desulfovibrio* genera with *Desulfovibrio* isolated from the human gut. JN-1~53 were from HV (Healthy volunteers); JN-B1~B35 were from LC (patients with liver cirrhosis). The dendrogram was constructed by Neighbor-Joining methods using almost complete 16S rRNA gene sequences. The scale indicates the genetic distance between the species. Blue star means isolates from HV, orange-red star means isolates from LC, and purple star means type strains of *Desulfovibrio* and *Cupidesulfovibrio*.

Next, we assembled a phylogenetic tree based on 16S rRNA gene sequence alignment (Figure 2). HV-source and LC-source *Desulfovibrio* species formed separated lineages. The HV isolates, *D. intestinalis* JN, *D. simplex* JN, *D. piger* JN, *D. legallii* JN, *C. oxamicus* JN, and *D. fairfieldensis* JN clustered with type strains. However, the LC isolates formed three major lineages: a cluster of *C. oxamicus* and two clusters of *D. desulfuricans*. The same species of *D. desulfuricans* were clustered into three main groups, including a group of HV-source, a group of LC-source, and a mixed group. In *C. oxamicus*, strains derived from HV and those from LC were divided into two groups. These data indicate that there were strain- and species-level differences between different sources of *Desulfovibrio*. Moreover, the LC-derived strains may have some specific mutations at the genetic level that enable them to be distinguished from the HV-derived strains in the analysis of 16S rRNA genes, which requires further investigation.

### 3.2. Desulfurization Performance Evaluation of Isolated Desulfovibrio Strains

A recent study indicates that *Desulfovibrio* may be affected by the concentration of Fe^2+^ in the culture system [19]. There was FeSO_4_ in the EDGM medium, and *Desulfovibrio* could reduce SO_4_^2−^ in the culture system and combine with Fe^2+^ to produce FeS that showed black precipitation, which might have impacted the detection of OD_600_, so we explored the effect of FeS suspension on OD_600_ detection in the concentration range (0.078125, 0.15625, 0.3125, 0.625, 1.25, 2.5, 5, 10 mM) of this study. The results showed that FeS in the concentration range of this study would not affect the detection of OD_600_ (Figure S1a). 

In the medium, SO_4_^2−^ can be reduced to H_2_S and FeS by *Desulfovibrio* at the same time, which will lead to a change in pH. So, the changes in pH and concentration of Fe^2+^ of the culture system were used as the evaluation index of *Desulfovibrio* desulfurization capacity. The results showed that when the concentration of Fe^2+^ was lower than 3.6 mM, the growth of *D. desulfuricans* ATCC 29577 was promoted with the increase of Fe^2+^ concentration, and the scavenging rate (desulfurization performance) of pH and SO_4_^2−^ in the culture system also increased (Figure S1b). When the concentration of Fe^2+^ is higher than 3.6 mM, the desulfurization performance of *D. desulfuricans* ATCC 29577 was also inhibited with the increase of Fe^2+^ concentration (Figure S1c). The above results showed that Fe^2+^ could promote the growth of *D. desulfuricans* ATCC 29577 and improve its desulfurization performance in a certain concentration range. Therefore, the concentration of Fe^2+^ (3.6 mM) was used for the subsequent experiment.

**Figure 3:**
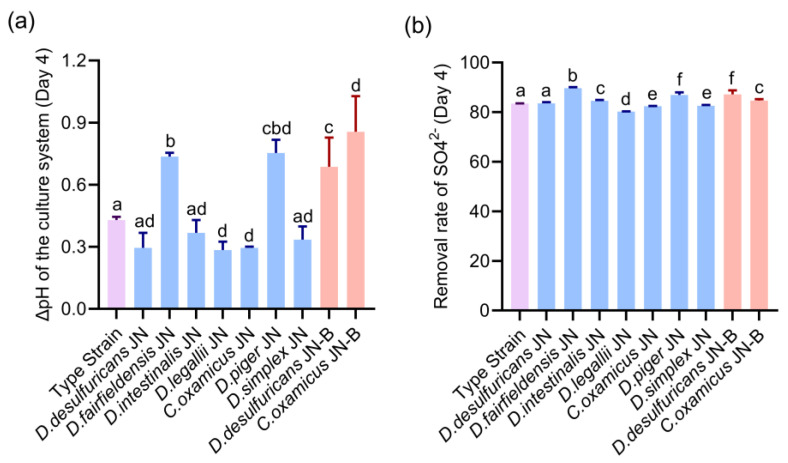
Desulfurization performance evaluation on *Desulfovibrio* from the human gut. (**a**) The change of the medium’s pH value from day 0 to day 4. (**b**) Comparison of SO_4_^2−^ removal rate. The type strain was *D. desulfuricans* ATCC 29577. The Blue bars were the strains from healthy volunteers, and the red bars were the stains from patients with liver cirrhosis, and the data are given as the means ± standard error of the mean (s.e.m.). Differences were identified using the *t*-test and One-way ANOVA statistical analysis, and different superscripts indicate significant differences (*p* < 0.05).

We observed that the pH of the medium of all isolated *Desulfovibrio* strains showed an upward trend after four days of culture (Figure 3a). The pH changes showed that in the *Desulfovibrio* derived from HV, the pH of *D. fairfieldensis* JN, *C. oxamicus* JN, *D. piger* JN, *D. desulfuricans* JN, and *D. legallii* JN was significantly higher than that of *D. desulfuricans* ATCC 29577 (Figure 3a). Among them, the pH of *D. fairfieldensis* JN and *D. piger* JN increased from 8.04 and 8.00 to 8.78 and 8.75, respectively (Table S3). The ΔpH of other *Desulfovibrio* (*D. intestinalis* JN and *D. simplex* JN) was not significantly different from that of the type strain. Compared with the *D. desulfuricans* JN strain from HV, all the *D. desulfuricans* JN-B from LC showed that the pH of the culture system increased significantly with the extension of time (Table S3). At the same time, compared with *C. oxamicus* JN, the strain *C. oxamicus* JN-B also showed a significant increase in ΔpH in the culture system (Table S3). 

In the detection of the SO_4_^2−^ clearance rate (Figure 3b), *Desulfovibrio* strains (*D. fairfieldensis* JN, *D. Legallii* JN, *C. oxamicus* JN, *D. piger* JN, and *D. simplex* JN) derived from HV showed significant difference compared with the standard strain *D. desulfuricans* ATCC 29577. Among them, the average SO_4_^2−^ clearance rate of *D. fairfieldensis* JN was significantly stronger than that of other species, which can reach about 89.69% on the fourth day. There was no significant difference in *D. desulfuricans* JN and *D. intestinalis* JN compared with the control group. On the other hand, the clearance rates of SO_4_^2−^ in *D. desulfuricans* JN-B (87.20%) and *C. oxamicus* JN-B (84.71%) derived were significantly different from that in the control group and *D. desulfuricans* JN (83.56%) and *C. oxamicus* JN (82.46%) derived from HV. Taken together, these data indicate that the desulfurization capacities of different strains of *Desulfovibrio* in normal human gut were different, and the *Desulfovibrio* isolated from the patients with LC had a higher SO_4_^2−^ clearance capacity than the same species isolated from HV.

### 3.3. Isolated Desulfovibrio Strains from Patients Have a Stronger Ability for Hydrogen Sulfide Production

**Figure 4:**
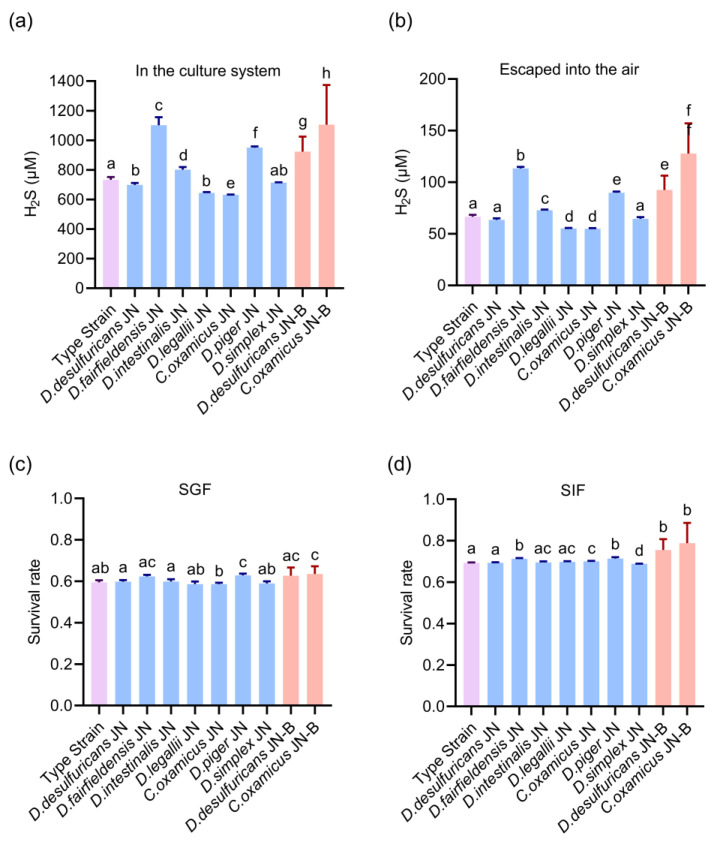
Hydrogen sulfide production and gastrointestinal tolerance of *Desulfovibrio* from the human gut. (**a**,**b**) The hydrogen sulfide production of isolates (**a**) in the culture system and (**b**) escaped into the air after 4 days of culture. (**c**,**d**) Evaluation of intestinal colonization of *Desulfovibrio* in (**c**) SGF (simulated gastric fluid) and (**d**) SIF (simulated intestinal fluid). The type strain was *D. desulfuricans* ATCC 29577. The blue bars were the strains from healthy volunteers, and the red bars were the stains from patients with liver cirrhosis, and the data are given as the means ± standard error of the mean (s.e.m.). Differences were identified using the t-test and One-way ANOVA statistical analysis, and different superscripts indicate significant differences (*p* < 0.05).

Hydrogen sulfide (H_2_S) is a toxic end-product of the growth and metabolism of *Desulfovibrio*, which can play a critical role in numerous biological functions. To compare the H_2_S production capacity of different *Desulfovibrio*, we measured two parts of H_2_S production, one in the culture system and the other dispersed into the air. The content of H_2_S in the culture system of *Desulfovibrio* derived from HV, including *D. fairfieldensis* JN and *D. piger* JN, was significantly higher than that of the type strain, which was about 1101.99 ± 51.20 μM and 950.14 ± 42.37 μM, respectively, after 4 days of culture (Figure 4a). In contrast, the content of H_2_S in the *C. oxamicus* JN culture system was lower than that in *D. desulfuricans* ATCC 29577 (about 637.31 ± 15.06 μM). Overall, the content of H_2_S in the medium of the *Desulfovibrio* from LC was significantly higher than that in *D. desulfuricans* ATCC 29577: up to 877.16 ± 13.53 μM (Figure 4a). Through comparison of the same strain, it was found that under the same conditions, the content of H_2_S produced by patient isolates (*D. desulfuricans* JN-B 926.02 ± 104.43 μM and *C. oxamicus* JN-B 1161.34 ± 281.61 μM) were significantly higher than that of HV (*D. desulfuricans* JN 697.67 ± 13.17 μM and *C. oxamicus* JN 630.60 ± 1.80 μM). 

At the same time, the dissolving amount of H_2_S produced by *D. desulfuricans* JN-B from LC was significantly higher than that of the same strain from HV (Figure 4b). It was observed that the content of escaping H_2_S in *Desulfovibrio* derived from HV was the highest in *D. fairfieldensis* JN and *D. piger* JN, which was significantly higher than that in the type strain, reaching 113.32 ± 1.29 μM and 89.77 ± 1.03 μM, respectively. Similar to the H_2_S content in the medium, *D. legallii* JN and *C. oxamicus* JN were also less than the control. The experimental results of LC showed that the H_2_S content of *D. desulfuricans* JN-B derived from LC was significantly higher than that of the standard strain, up to 87.82 ± 2.14 μM. Moreover, the proportion of H_2_S escape of all isolates was between 8.00%~10.00% (Figure 4b). The above results showed that the H_2_S production capacities of *D. fairfieldensis* and *D. piger* were the strongest compared with other *Desulfovibrio* species in this study, *D. intestinalis* was the second, and *D. legallii* and *C. oxamicus* were the weakest. These data also suggest that the H_2_S production ability of the strains from patients with liver cirrhosis was significantly stronger than that of healthy volunteers.

### 3.4. Gastrointestinal Tolerance of Isolated Desulfovibrio Strains

To analyze the tolerance of the different *Desulfovibrio* in the human gut, we used two artificial gastrointestinal fluids. The tolerance of *Desulfovibrio* isolated from healthy people and patients’ intestines was similar to that of *D. desulfuricans* ATCC 29577 in SIF and SGF. However, there were significant differences in tolerance between *D. desulfuricans* JN-B from LC and *D. desulfuricans* JN from HV in SIF and SGF (Figure 4c,d). The survival rates of *D. desulfuricans* JN-B and *C. oxamicus* JN-B in SGF increased by 2.898% and 4.848%, respectively. In SIF, the survival rate of *D. desulfuricans* JN-B increased by 6.210%, and *C. oxamicus* JN-B increased by 5.932%. These results suggest that the gastrointestinal tolerance of *Desulfovibrio* strains from patients with liver cirrhosis was harder than that of healthy volunteers.

### 3.5. The Desulfovibrio Isolates from the Patients with Cirrhosis Are More Resistant to Antibiotics

Antibiotic resistance plays a vital role in bacteria culture and clinical selection. Some studies have identified the susceptibilities to antibiotics of *D. desulfuricans* and *D. piger* [28,29], but other *Desulfovibrio* including *D. simplex* and *D. intestinalis* have not been measured. Moreover, the antibiotic sensitivity of *Desulfovibrio* in the intestines of patients may change after clinical treatment. Several common antibiotics (Neomycin sulphate, Polymyxin B sulphate, Nisin, Ampicillin, Metronidazole, and Kanamycin sulphate) were examined in these isolates in comparison to *D. desulfuricans* ATCC 29577. Our results showed that *Desulfovibrio* was resistant to many common antibiotics, such as neomycin sulfate and nisin (Table 1, Figure S2). For polymyxin B sulphate, the isolated strains showed a certain sensitivity compared with other antibiotics, and the growth of strains was partially inhibited, but there was no significant differences among different strains. Additionally, all *Desulfovibrio* strains were sensitive to kanamycin sulphate and metronidazole. Some species of *Desulfovibrio* showed ampicillin sensitivity, but *D. desulfuricans* was not sensitive to ampicillin. *D. fairfieldensis* was between other *Desulfovibrio* and *D. desulfuricans* in sensitivity to ampicillin (Table 1, Figure S2). 

For *Desulfovibrio* strains from LC, their resistance to all antibiotics increased in varying degrees. Among them, the resistance of *C. oxamicus* JN-B21 and *C. oxamicus* JN-B29 to ampicillin increased significantly, and their MIC values were 45 μg/mL and 40 μg/mL, respectively (Table 2). The LC isolates *D. desulfuricans* JN-B13, *D. desulfuricans* JN-B18, and *C. oxamicus* JN-B29 displayed much higher MIC values for kanamycin sulphate, which was 30 μg/mL. The resistance level to metronidazole appeared similar for LC isolates, ranging from 2.5 μg/mL to 5.0 μg/mL (Table 2). From the above results, it can be seen that *Desulfovibrio* has species-level differences in resistance to the same type of antibiotics, and the *Desulfovibrio* isolated from patients with liver cirrhosis had increasing antibiotic resistance.

## 4. Discussion

Despite the fact that *Desulfovibrio* is a known microorganism associated with a variety of human diseases, there is still little information about the changes of *Desulfovibrio* in patients with hepatic cirrhosis. Liver cirrhosis can be a consequence of NAFLD, hepatitis B or C infection, high alcohol consumption, etc. In addition, most of the studies remain at the level of family and genus, and the differences at the species level have not been deeply explored. As some studies have described [12,13], the physiological effects of different species may differ. Therefore, it is of great significance to explore the differences of different *Desulfovibrio* in the guts of patients and healthy people to analyze its mechanism of action. In this study, we obtained a total of 88 strains of *Desulfovibrio* from the human gut, and there were strain- and species-level differences. To our knowledge, this is the first time that *D. intestinalis* and *D. simplex* have been isolated from the human intestinal tract. *D. desulfuricans* was the dominant species in isolates from both HV and LC. In addition, we found *Desulfovibrio* isolated from the patients with LC generally had a higher desulfurization capability, H_2_S production capacity, intestinal colonization capability, and levels of antibiotic resistance than the same species isolated from HV.

Previous studies demonstrated that spectrophotometry was usually used to detect the H_2_S yield of strains. However, this method had some limitations in that it could not capture the H_2_S escaping from the culture medium to the air. Therefore, we combined the spectrophotometry and a 96-well plate method for the rapid detection of H_2_S gas molecules to observe the H_2_S production. Increased intestinal permeability has been proposed as one of the main pathogenic mechanisms of NAFLD [30], and high levels of H_2_S are genotoxic to intestinal epithelium [31]. So *Desulfovibrio* might play a role in liver cirrhosis, which may be related to its ability to produce H_2_S.

Based on the evaluation of the tolerance of our isolated strains to SGF and SIF, LC-derived *Desulfovibrio* is more resistant to SIF than HV-derived *Desulfovibrio*, so they might be easier to colonize and proliferate in the intestines, which may be related to the increased abundance of *Desulfovibrio* in the intestines of patients with liver diseases [12]. 

Nowadays, antibiotics are often used as adjuvant therapy for digestive tract diseases. For the clinical treatment of anaerobic infections that may involve *Desulfovibrio*, more information on the antibiogram of *Desulfovibrio* is necessary. The research on the antibiotics sensitivity of *Desulfovibrio* was limited in strain types [28,29], the information on *D. simplex* and *D. intestinalis* is lacking, and the differences between strains among different individuals had not been explored. Our antibiotic sensitivity test shows that *Desulfovibrio* is resistant to most antibiotics, so the commonly used drugs could not achieve the desired effect. Among them, *D. desulfuricans* was resistant to ampicillin, and the tolerance of *D. fairfieldensis* to ampicillin was stronger than that of other strains (Figure S2). This is because ampicillin is a β-lactam antibiotic, whereas *D. fairfieldensis* is less sensitive to β-lactam. *D. desulfuricans* is not sensitive to β-lactam because it can produce β-lactamase [21]. Moreover, compared with the isolates from HV, the isolated bacteria from patients showed stronger antibiotic resistance. About 50–60% of them tested in this study developed resistance to the ampicillin and Kanamycin sulphate. Due to the genetic material detection from the prophages within the genomic sequence [32,33], this property may be related to the horizontal transfer of viral genes. 

Furthermore, the differences in other physiological characteristics also indicate the potentially diverse roles that *Desulfovibrio* plays in human health. What needs to be pointed out is that our estimated coverages of strains can only indicate the present probabilities of corresponding strains in samples. More experiments need to be conducted to the hypothesis of the promoting effect of *Desulfovibrio* on liver cirrhosis. Nonetheless, the gained results of the research represent useful information for exploring the relationship and influence mechanism between *Desulfovibrio* and liver cirrhosis or related diseases at the molecular level and community level in the future, *Desulfovibrio* may be a potential biotherapeutic target for the treatment of liver disease.

## 5. Conclusions

In this study, 88 strains of *Desulfovibrio* were isolated and identified from healthy people and patients with hepatic cirrhosis. Our results suggest that there was a significant difference in the desulfurization ability and the H_2_S production ability of the different species of HV-derived *Desulfovibrio*. LC-derived *Desulfovibrio* showed stronger hydrogen sulfide production ability, stronger gastrointestinal tolerance, and higher antibiotic resistance compared with the HV-derived *Desulfovibrio*. These results show that the growth and metabolism were different among various *Desulfovibrio* species, and there are many differences between *Desulfovibrio* in the intestines of patients with liver cirrhosis and healthy people. The results also provide a theoretical basis for the subsequent exploration of the relationship between *Desulfovibrio* and the development of liver cirrhosis.

## Figures and Tables

**Table 1:** Sensitivity of Desulfovibrio from the human gut to different antibiotics. S—Susceptible, Standard dosing regimen; I—Susceptible, Increased exposure; R—Resistance (Data reference EUCAST).

Strain	Neomycin Sulphate	Polymyxin B Sulphate	Nisin	Ampicillin	Metronidazole	Kanamycin Sulphate
*D. desulfuricans* ATCC 29577	R	R	R	R	S	S
*D. desulfuricans* JN-1	R	R	R	R	S	S
*D. desulfuricans* JN-16	R	R	R	R	S	S
*D. desulfuricans* JN-33	R	R	R	R	S	S
*D. desulfuricans* JN-35	R	R	R	R	S	S
*D. fairfieldensis* JN-45	R	R	R	R	S	S
*D. intestinalis* JN-4	R	R	R	I	S	S
*D. intestinalis* JN-50	R	R	R	I	S	S
*D. legallii* JN-32	R	R	R	S	S	S
*C. oxamicus* JN-14	R	R	R	I	S	S
*D. piger* JN-20	R	R	R	I	S	S
*D. simplex* JN-24	R	R	R	I	S	S
*D. desulfuricans* JN-B5	R	R	R	R	S	S
*D. desulfuricans* JN-B13	R	R	R	R	S	I
*D. desulfuricans* JN-B17	R	R	R	R	S	S
*D. desulfuricans* JN-B18	R	R	R	R	S	I
*C.oxamicus* JN-B21	R	R	R	R	R	S
*C.oxamicus* JN-B24	I	R	R	R	R	S
*C.oxamicus* JN-B27	I	R	R	R	S	S
*C.oxamicus* JN-B29	R	R	R	R	S	I

**Table 2:** The minimum inhibitory concentration (MIC) of antibiotics for Desulfovibrio from the human gut.

Strain	MIC (μg/mL)		
	Ampicillin	Kanamycin Sulphate	Metronidazole
*D. desulfuricans* ATCC 29577	\	0.000	0.750
*D. desulfuricans* JN-1	\	0.000	0.750
*D. intestinalis* JN-4	1.000	0.000	1.250
*C. oxamicus* JN-14	0.750	0.750	1.000
*D. piger* JN-20	1.250	0.000	1.000
*D. simplex* JN-24	1.000	0.250	0.750
*D. legallii* JN-32	0.125	0.500	0.250
*D. fairfieldensis* JN-45	\	0.000	1.250
*D. desulfuricans* JN-B5	\	0.750	2.500
*D. desulfuricans* JN-B13	\	30.00	2.500
*D. desulfuricans* JN-B17	\	2.500	2.500
*D. desulfuricans* JN-B18	\	30.00	2.500
*C.oxamicus* JN-B21	45	2.5	5.000
*C.oxamicus* JN-B24	2.5	1.0	5.000
*C.oxamicus* JN-B27	2.5	1.25	2.500
*C.oxamicus* JN-B29	40	30	2.500

## Data Availability

Not applicable.

## Supplementary Materials

The following supporting information can be downloaded at: https://www.mdpi.com/article/10.3390/microorganisms11020276/s1, Table S1: Information of healthy volunteers (HV) and patients with liver cirrhosis (LC); Table S2: Summary of information of *Desulfovibrio* from human gut; Table S3: The minimum inhibitory concentration (MIC) of antibiotics for *Desulfovibrio* from human gut; Figure S1: Effect of medium composition on Desulfurization performance Test; Figure S2: Sensitivity of *Desulfovibrio* from human gut to different antibiotics.
