# Supplementary material for: Diversity and Comparison of Intestinal Desulfovibrio in Patients with Liver Cirrhosis and Healthy People
Source: Microorganisms. 2023 Jan 20;11(2):276. doi: 10.3390/microorganisms11020276 (PMC9960842; doi:10.3390/microorganisms11020276)
Supplement: Supplementary file 1 [file microorganisms-11-00276-s001.zip › microorganisms-2141803-supplementary.pdf]

Supplementary Table S1: Information of healthy volunteers (HV) and patients with liver cirrhosis (LC).

Sample ID	Country	Gender	Age	BMI (kg/m2)	Cirrhotic (Y or N)	HBV related (Y or N)	INR	Crea (μmol/L)	Alb (g/L)	TB (μmol/L)	PLT (10^9/L)	PT (S)	Ascites
LC-1	China	male	52	20	Y	N	1.21	53	32.5	377	37	13.5	Mild
LC-2	China	male	53	19	Y	N	1.05	62	39.4	127	44	16.7	Mild
LC-3	China	male	34	22	Y	N	1.33	51	26.1	81	56	13.5	None
LC-4	China	male	44	23	Y	N	1.25	77	30.5	122	61	19.6	None
LC-5	China	male	43	21	Y	N	1.15	65	23.7	177	43	13.5	None
LC-6	China	male	51	21	Y	N	2.03	74	22.6	150	40	12.4	None
LC-7	China	male	83	23	Y	N	1.08	487	29.9	10.8	76	12.5	Medium
LC-8	China	male	51	22	Y	Y	1.34	56	30	47.2	57	15.5	Heavy
LC-9	China	female	77	29	Y	N	1.16	55.6	28.1	26.6	51	13.5	Mild
HV-1	China	male	27	19	N	N	1.35	57	39.4	5.10	139	12.4	None
HV-2	China	male	27	24	N	N	1.34	49	42.0	5.59	164	12.9	None
HV-3	China	male	27	22	N	N	1.15	62	41.7	8.28	138	12.8	None
HV-4	China	male	25	24	N	N	1.02	54	36.2	8.40	170	11.8	None
HV-5	China	female	24	18	N	N	1.14	43	47.4	4.27	105	11.2	None
HV-6	China	female	24	17	N	N	1.15	40	44.2	5.73	167	11.7	None

Supplementary Table S2: Summary of information of *Desulfovibrio* from human gut. Strains JN-1~53 were from Healthy Volunteer, and strains JN-B1~B35 were from patients with Liver Cirrhosis.

Strain Number	GenBank Accession No.	Percent Identity	Sequence Length(bp)
Desulfovibrio desulfuricans JN-1	NR_104990.1	99.79%	1542
Desulfovibrio desulfuricans JN-2	NR_104990.1	99.72%	1542
Desulfovibrio intestinalis JN-3	NR_026413.1	100.00%	1509
Desulfovibrio intestinalis JN-4	NR_026413.1	100.00%	1509
Cupidesulfovibrio oxamicus JN-5	NR_043567.1	100.00%	1513
Desulfovibrio desulfuricans JN-6	NR_104990.1	99.72%	1542
Desulfovibrio intestinalis JN-7	NR_026413.1	100.00%	1509
Desulfovibrio desulfuricans JN-8	NR_104990.1	99.72%	1542
Desulfovibrio intestinalis JN-9	NR_026413.1	100.00%	1509
Desulfovibrio desulfuricans JN-10	NR_104990.1	96.82%	1542
Desulfovibrio desulfuricans JN-11	NR_104990.1	99.58%	1542
Desulfovibrio piger JN-12	NR_041778.1	100.00%	1542
Desulfovibrio desulfuricans JN-13	NR_104990.1	99.30%	1542
Cupidesulfovibrio oxamicus JN-14	NR_043567.1	100.00%	1513
Desulfovibrio desulfuricans JN-15	NR_104990.1	99.45%	1542
Desulfovibrio desulfuricans JN-16	NR_104990.1	99.79%	1542
Desulfovibrio simplex JN-17	NR_117110.1	100.00%	1539
Desulfovibrio piger JN-18	NR_041778.1	100.00%	1542
Desulfovibrio simplex JN-19	NR_117110.1	100.00%	1539
Desulfovibrio piger JN-20	NR_041778.1	99.87%	1542
Desulfovibrio piger JN-21	NR_041778.1	99.87%	1542
Desulfovibrio desulfuricans JN-22	NR_104990.1	99.38%	1542
Desulfovibrio intestinalis JN-23	NR_026413.1	100.00%	1509
Desulfovibrio simplex JN-24	NR_113296.1	100.00%	1539
Desulfovibrio desulfuricans JN-25	NR_104990.1	99.31%	1542
Desulfovibrio simplex JN-26	NR_113296.1	100.00%	1539
Desulfovibrio desulfuricans JN-27	NR_104990.1	99.79%	1542
Desulfovibrio desulfuricans JN-28	NR_104990.1	99.79%	1542
Desulfovibrio desulfuricans JN-29	NR_104990.1	99.72%	1542

Desulfovibrio desulfuricans JN-30	NR_104990.1	97.25%	1542
Desulfovibrio desulfuricans JN-31	NR_104990.1	99.58%	1542
Desulfovibrio legallii JN-32	NR_108301.1	100.00%	1443
Desulfovibrio desulfuricans JN-33	NR_104990.1	99.79%	1542
Desulfovibrio desulfuricans JN-34	NR_104990.1	99.65%	1542
Desulfovibrio desulfuricans JN-35	NR_104990.1	99.65%	1542
Desulfovibrio simplex JN-36	NR_113296.1	100.00%	1539
Desulfovibrio intestinalis JN-37	NR_026413.1	100.00%	1509
Desulfovibrio desulfuricans JN-38	NR_104990.1	99.72%	1542
Desulfovibrio desulfuricans JN-39	NR_104990.1	99.72%	1542
Desulfovibrio legallii JN-40	NR_108301.1	99.86%	1443
Desulfovibrio desulfuricans JN-41	NR_104990.1	99.72%	1542
Desulfovibrio desulfuricans JN-42	NR_104990.1	99.72%	1542
Desulfovibrio desulfuricans JN-43	NR_104990.1	99.65%	1542
Desulfovibrio piger JN-44	NR_041778.1	99.87%	1542
Desulfovibrio fairfieldensis JN-45	NR_104990.1	99.03%	1455
Desulfovibrio desulfuricans JN-46	NR_104990.1	99.72%	1542
Desulfovibrio simplex JN-47	NR_113296.1	100.00%	1539
Desulfovibrio desulfuricans JN-48	NR_104990.1	99.72%	1542
Desulfovibrio intestinalis JN-49	NR_026413.1	100.00%	1509
Desulfovibrio intestinalis JN-50	NR_026413.1	100.00%	1509
Desulfovibrio desulfuricans JN-51	NR_104990.1	99.72%	1542
Desulfovibrio desulfuricans JN-52	NR_104990.1	99.65%	1542
Desulfovibrio desulfuricans JN-53	NR_104990.1	99.72%	1542
Desulfovibrio desulfuricans JN-B1	NR_104990.1	99.93%	1542
Desulfovibrio desulfuricans JN-B2	NR_104990.1	100.00%	1542
Desulfovibrio desulfuricans JN-B3	NR_104990.1	99.67%	1542
Desulfovibrio desulfuricans JN-B4	NR_104990.1	99.87%	1542
Desulfovibrio desulfuricans JN-B5	NR_104990.1	100.00%	1542
Desulfovibrio desulfuricans JN-B6	NR_104990.1	100.00%	1542
Desulfovibrio desulfuricans JN-B7	NR_104990.1	99.87%	1542
Desulfovibrio desulfuricans JN-B8	NR_104990.1	100.00%	1542
Desulfovibrio desulfuricans JN-B9	NR_104990.1	99.93%	1542
Desulfovibrio desulfuricans JN-B10	NR_104990.1	99.86%	1542

Desulfovibrio desulfuricans JN-B11	NR_104990.1	99.72%	1542
Desulfovibrio desulfuricans JN-B12	NR_104990.1	99.58%	1542
Desulfovibrio desulfuricans JN-B13	NR_104990.1	99.29%	1542
Desulfovibrio desulfuricans JN-B14	NR_104990.1	99.58%	1542
Desulfovibrio desulfuricans JN-B15	NR_104990.1	99.31%	1542
Desulfovibrio desulfuricans JN-B16	NR_104990.1	99.22%	1542
Desulfovibrio desulfuricans JN-B17	NR_104990.1	99.65%	1542
Desulfovibrio desulfuricans JN-B18	NR_104990.1	99.22%	1542
Desulfovibrio desulfuricans JN-B19	NR_104990.1	99.22%	1542
Desulfovibrio desulfuricans JN-B20	NR_104990.1	99.51%	1542
Desulfovibrio desulfuricans JN-B21	NR_104990.1	99.51%	1542
Cupidesulfovibrio oxamicus JN-B22	NR_043567.1	98.58%	1513
Cupidesulfovibrio oxamicus JN-B23	NR_043567.1	98.57%	1513
Cupidesulfovibrio oxamicus JN-B24	NR_043567.1	99.02%	1513
Cupidesulfovibrio oxamicus JN-B25	NR_043567.1	99.16%	1513
Cupidesulfovibrio oxamicus JN-B26	NR_043567.1	98.69%	1513
Cupidesulfovibrio oxamicus JN-B27	NR_043567.1	98.96%	1513
Cupidesulfovibrio oxamicus JN-B28	NR_043567.1	99.58%	1513
Cupidesulfovibrio oxamicus JN-B29	NR_043567.1	99.31%	1513
Cupidesulfovibrio oxamicus JN-B30	NR_043567.1	98.38%	1513
Cupidesulfovibrio oxamicus JN-B31	NR_043567.1	99.10%	1513
Cupidesulfovibrio oxamicus JN-B32	NR_043567.1	99.31%	1513
Cupidesulfovibrio oxamicus JN-B33	NR_043567.1	99.44%	1513
Cupidesulfovibrio oxamicus JN-B34	NR_043567.1	99.58%	1513
Cupidesulfovibrio oxamicus JN-B35	NR_043567.1	99.44%	1513

Supplementary Table S3: The change in the medium's pH value from day 0 to day 6 of *Desulfovibrio* from human gut. The type strain was *D. desulfuricans* ATCC 29577. The data are given as the means ± standard error of the mean (s.e.m.).

Strain Number	Day 0	Day 1	Day 2	Day 3	Day 4	Day 5	Day 6
Type Strain	7.805 ± 0.006	7.923 ± 0.021	8.002 ± 0.017	8.173 ± 0.006	8.243 ± 0.005	8.23 ± 0.014	8.266 ± 0.009
D. desulfuricans JN	7.800 ± 0.008	7.926 ± 0.019	8.061 ± 0.012	8.150 ± 0.012	8.207 ± 0.004	8.21 ± 0.011	8.207 ± 0.010
D. fairfieldensis JN	7.800 ± 0.008	8.040 ± 0.022	8.257 ± 0.033	8.693 ± 0.005	8.777 ± 0.005	8.78 ± 0.014	8.770 ± 0.008
D. intestinalis JN	7.803 ± 0.005	7.943 ± 0.018	8.092 ± 0.018	8.195 ± 0.013	8.262 ± 0.004	8.264 ± 0.011	8.262 ± 0.008
D. legallii JN	7.800 ± 0.008	7.961 ± 0.019	8.166 ± 0.018	8.383 ± 0.015	8.456 ± 0.014	8.455 ± 0.010	8.456 ± 0.012
C. oxamicus JN	7.803 ± 0.005	7.941 ± 0.020	8.115 ± 0.018	8.263 ± 0.015	8.333 ± 0.011	8.332 ± 0.009	8.331 ± 0.012
D. piger JN	7.797 ± 0.005	7.978 ± 0.020	8.187 ± 0.021	8.430 ± 0.016	8.510 ± 0.010	8.507 ± 0.010	8.511 ± 0.011
D. simplex JN	7.803 ± 0.009	7.975 ± 0.020	8.191 ± 0.02	8.440 ± 0.015	8.518 ± 0.014	8.516 ± 0.010	8.518 ± 0.012
D.desulfuricans JN-B	7.800 ± 0.007	7.968 ± 0.030	8.190 ± 0.082	8.326 ± 0.088	8.452 ± 0.155	8.513 ± 0.185	8.557 ± 0.137
C. oxamicus JN-B	7.776 ± 0.062	7.942 ± 0.040	8.456 ± 0.047	8.487 ± 0.084	8.591 ± 0.067	8.637 ± 0.055	8.657 ± 0.066

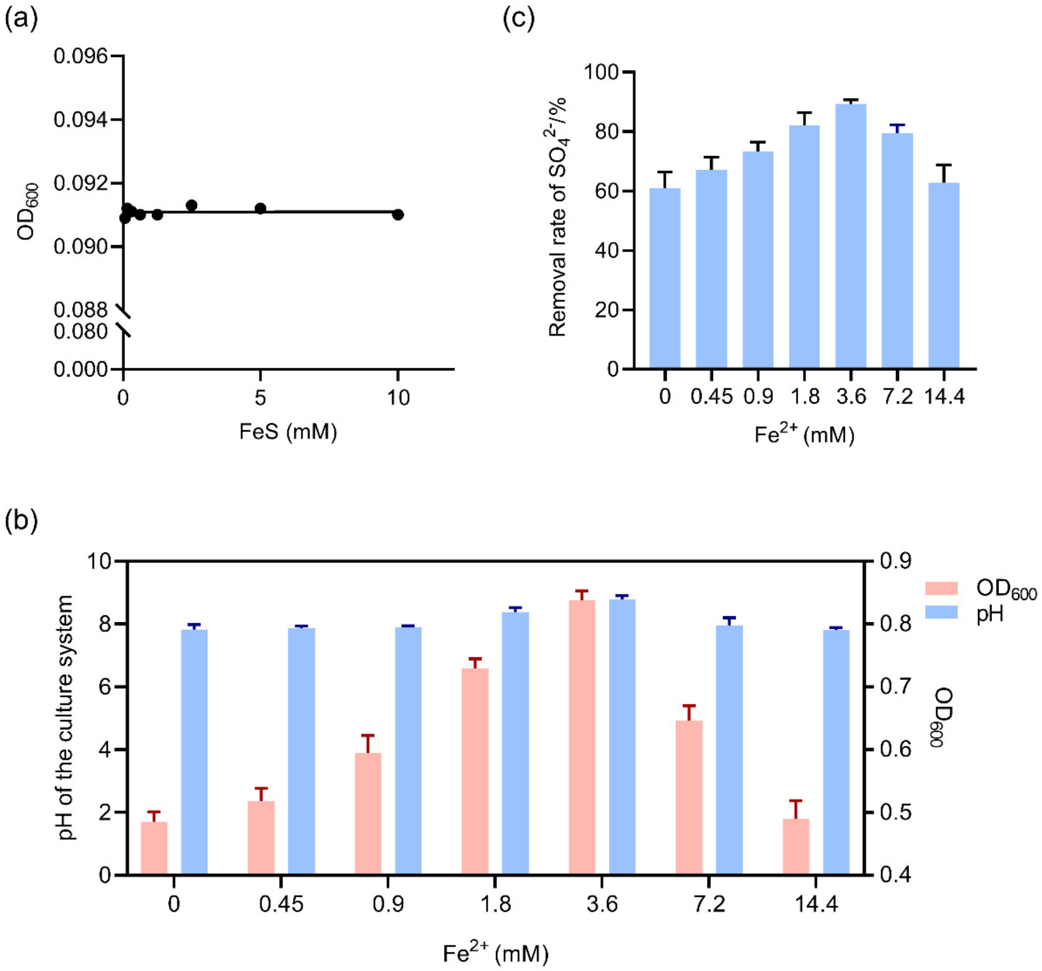

Supplementary Figure S1: Effect of Fe^{2+} concentration in the medium on *D. desulfuricans* ATCC 29577. (a) The relationship of FeS concentration in medium with OD_{600} of *D. desulfuricans* ATCC 29577. (b) The pH in the culture system and OD_{600} of *D. desulfuricans* ATCC 29577 under different Fe^{2+} concentrations. (c) The SO_4^{2-} removal rate of *D. desulfuricans* ATCC 29577 under different Fe^{2+} concentrations.

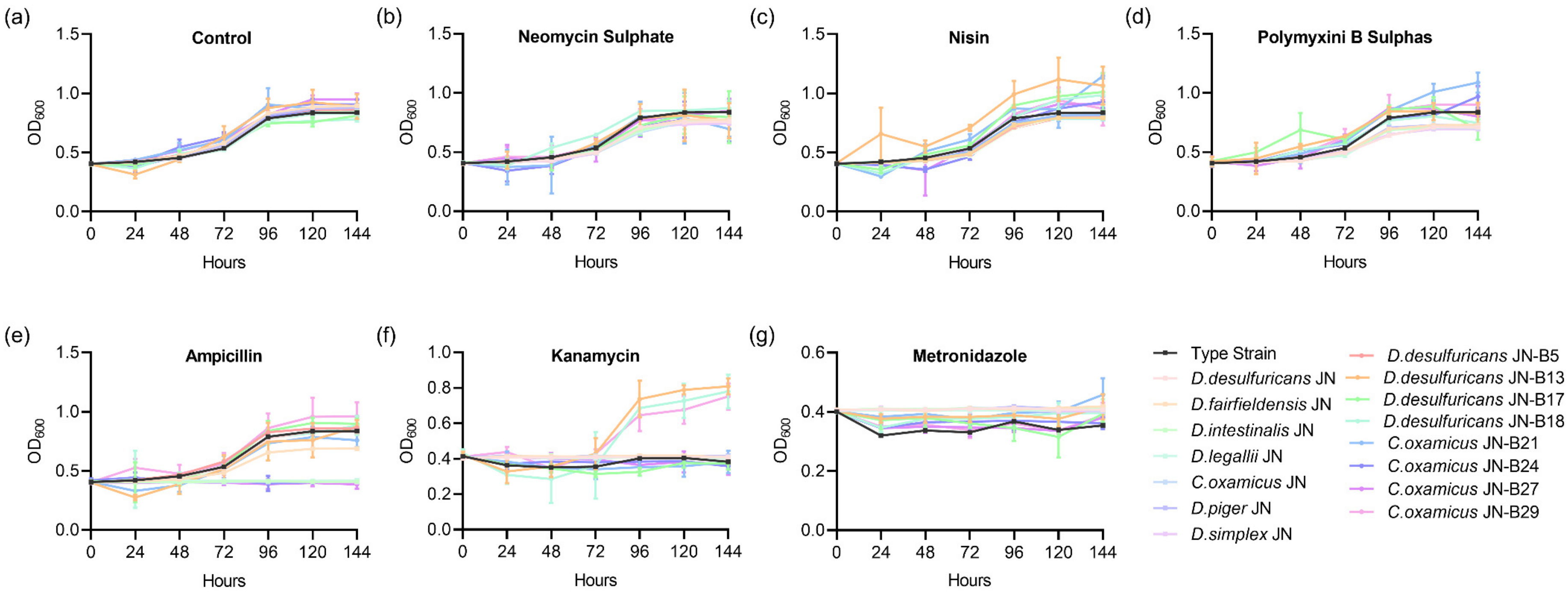

Supplementary Figure S2: Antibiotic sensitivity of type strain and different strains of *Desulfovibrio* from the human gut. (a) Control (without antibiotics). (b) Neomycin sulphate (40 μg/mL). (c) Nisin (25 μg/mL). (d) Polymyxin B sulphate (25 μg/mL). (e) Ampicillin (25 μg/mL). (f) Metronidazole (20 μg/mL). (g) Kanamycin sulphate (20 μg/mL). Type strain: *D. desulfuricans* ATCC 29577.
